# Use of the Brazilian People’s Pharmacy Program by older adults

**DOI:** 10.1590/S1518-8787.2016050006180

**Published:** 2016-04-15

**Authors:** Vanessa Iribarrem Avena Miranda, Anaclaudia Gastal Fassa, Rodrigo Dalke Meucci, Bárbara Heather Lutz

**Affiliations:** I Programa de Pós-Graduação em Epidemiologia. Faculdade de Medicina. Universidade Federal de Pelotas. Pelotas, RS, Brasil; II Departamento de Medicina Social. Faculdade de Medicina. Universidade Federal de Pelotas. Pelotas, RS, Brasil

**Keywords:** Aged, Community Pharmacy Services, Equity in Access, Drug Utilization, Pharmacoepidemiology, Cross-Sectional Studies

## Abstract

**OBJECTIVE:**

To assess the prevalence and factors associated with the use of the expanded Brazilian People’s Pharmacy Program among older adults and the reasons for not using it.

**METHODS:**

In this population-based cross-sectional study conducted in the urban area of Pelotas, RS, Southern Brazil, we evaluated 1,305 older adults (aged 60 years or over) who had used medication in the last 15 days. Independent variables were socioeconomic factors, economic status, household income in minimum wages, educational attainment in years of schooling and occupational status. Demographic variables were sex, age, marital status, and self-reported skin color/race. Poisson regression was employed to analyze the factors associated with the use of the program.

**RESULTS:**

The prevalence of use was 57.0% whilst the prevalence of knowledge of the program was 87.0%. In individuals aged 80 years or over, use of the program was 41.0%. As to the origin of the prescriptions used by older adults, 46.0% were from the Brazilian Unified Health System. The main reasons for not using the program were: difficulty in getting prescriptions, medication shortage, and ignorance about the medications offered and about the program. Higher age, lower income, presence of chronic diseases, and use of four or more medications were associated with use of the program.

**CONCLUSIONS:**

It is necessary to expand the knowledge and use of the Brazilian People’s Pharmacy Program, especially among older adults, and to improve the dissemination of its list of medications to users and physicians. Thus it will be possible to reduce spending on long-term medications, which are especially important for this population.

## RESUMO

**OBJETIVO:**

Avaliar a prevalência e os fatores associados à utilização do Programa Farmácia Popular do Brasil – modelo expansão entre idosos e os motivos de não utilização.

**MÉTODOS:**

Neste estudo transversal de base populacional, realizado na zona urbana de Pelotas, RS, foram avaliados 1.305 idosos (60 anos ou mais) que utilizaram medicamentos nos últimos 15 dias. Como variáveis independentes foram caracterizados fatores socioeconômicos, nível econômico, renda familiar em salários mínimos, escolaridade em anos de estudo e *status* ocupacional. As variáveis demográficas foram sexo, idade, situação conjugal e cor da pele autorreferida. Para análise dos fatores associados à utilização do PFPB-E, utilizou-se regressão de Poisson.

**RESULTADOS:**

A prevalência de utilização do programa foi de 57,0% e a de conhecimento foi de 87,0%. Nos indivíduos com 80 anos ou mais, a utilização do programa foi de 41,0%. Quanto à origem das prescrições utilizadas pelos idosos, 46,0% eram oriundas do SUS. Os principais motivos para não utilização foram: dificuldade em conseguir receita, falta do medicamento, desconhecimento sobre os medicamentos ofertados e sobre o programa. Maior idade, menor renda, ter doença crônica e usar quatro ou mais medicamentos estiveram associados à utilização.

**CONCLUSÕES:**

É preciso ampliar o conhecimento e a utilização do Programa Farmácia Popular do Brasil, especialmente entre os mais idosos, e melhorar a divulgação da lista de medicamentos do programa aos usuários e aos médicos. Dessa forma, será possível reduzir gastos com medicamentos de uso contínuo, que são especialmente importantes para essa população.

## INTRODUCTION

Ensuring access to basic and essential medications to all individuals is a priority in today’s health policies^13^ ^,^ ^a^. The Federal Government launched in 2004 the *Programa Farmácia Popular do Brasil* (PFPB – Brazilian People’s Pharmacy Program) as part of the Brazilian Unified Health System (SUS)^b^ ^,^ ^c^ ^,^ ^d^. This strategy aims to promote the expansion of access to medication and equal service^b^ to the entire population. The purpose is to avoid treatment quitting, especially by individuals with low income who use private health services, but have difficulty in buying the required medications in regular drugstores^20^ ^,^ ^e^.

The PFPB develops two axes of action: its own network of people’s pharmacies and the *Aqui tem Farmácia Popular* (People’s Pharmacy Here) extended network (PFPB-E). People’s Pharmacies, in operation since 2004, dispense 112 medications at cost value, representing a reduction of up to 90.0% of the market value. The PFPB-E, considered an expansion of the PFPB in partnership with private pharmacies and drugstores, was created with the goal of broadening the coverage of pharmaceutical assistance, promoting the integrality of health care^5^. In this mode, the Ministry of Health (MS) subsidizes 90.0% of the reference value for medication for diseases such as dyslipidemia, Parkinson’s disease, glaucoma, osteoporosis and rhinitis, in addition to birth control and adult diapers^f^. In 2011, with the creation of the *Saúde Não Tem Preço* (Health is Priceless) program, the two axes of action began dispensing free medication for asthma, diabetes and hypertension^g^.

Although the program is directed to all age groups, it is especially important for older adults, who tend to have bigger health needs, resulting in increased use of health services and medications^5^ ^,^ ^19^ ^,^ ^h^. In this sense, the PFPB-E helps control and prevent chronic diseases, increasing the population’s access to medical therapy and reducing the impact of medication cost on family budgets^3^.

As the PFPB is relatively new, studies about it are scarce and give greater emphasis to its own network, which was consolidated first. They are restricted to describing the program in terms of medication availability compared with the public and private sectors and the profile of the population that uses the program^8^ ^,^ ^14^ ^,^ ^15^. Although this study addresses the PFPB only on the private network, since there are no program units in the city of Pelotas, this is the first population-based study to obtain information on the use of the PFPB-E for each medication^i^ ^,^ ^j^.

Considering the importance of the universalization of access to medications for population health and the role of the PFPB to expand access, we aimed to assess the prevalence and factors associated with the use of the PFPB-E among older adults and the reasons for not using it.

## METHODS

This population-based cross-sectional study was performed with older adults (aged 60 years or over) in the urban area of Pelotas, RS, Southern Brazil. There are approximately 46,099 older adults in the urban area of this city (2010 Demographic Census, IBGE)^k^. According to data from the local health surveillance, there are about 165 pharmacies and drugstores in Pelotas, of which 62.0% are linked to the PFPB-E. This study is part of research “Avaliação da saúde de idosos da cidade de Pelotas”, conducted in the first semester of 2014.

The sample size estimation to study the prevalence of the use of the PFPB-E considered the following parameters: 95% confidence level, 60.0% estimated prevalence, tolerable error of four percentage points, and an increase of 10.0% for losses and refusals. To assess the factors associated with the outcome, the parameters used were: 95% confidence level, 80.0% minimum statistical power, 1:5 ratio between unexposed and exposed, 48.0% estimated prevalence for the unexposed, 1.3 prevalence ratio, an increase of 15.0% for confounding factors, and 1.5 design effect, totaling an estimated sample size of 1,246 older adults.

Sample selection was performed in two stages. In the first, the 488 census districts of Pelotas were listed and sorted according to average income, with a random sampling of 133 districts. This strategy ensured the inclusion of several neighborhoods of different economic status. In the second stage, occupied households in the selected districts were counted, estimating about one older adult for every three households. We drew 31 households by district, with the aim of reducing design effects, and sampled approximately 10 households in each selected district, totaling 4,123 households. All people aged 60 years or over in each household were invited to participate in the study. We excluded those living in nursing homes, unable to answer the questionnaire for mental or physical issues, and who lacked a companion or caregiver.

The outcome “use of the PFPB” was defined as obtaining at least one medication from the PFPB among older adults who had used any medication in the last 15 days. “Knowledge of the PFPB” was checked by answering yes to the question “Do you know the People’s Pharmacy Program?”

When respondents reported not obtaining the medication from the PFPB-E or SUS pharmacies (municipal or primary health care units), they were asked if they had tried to. In case of an affirmative answer, they were asked the reasons for failing to get the medication and, in case of a negative answer, why they had not sought the PFPB-E. The packaging or prescription of every medication used was requested to verify the correct names and later classify them by pharmacological groups. To trace the origin of prescriptions, the following question was made: “Who prescribed this medication for you?” (SUS physician or dentist; private or health plan physician or dentist).

The independent variables were: socioeconomic factors; economic status, classified according to the Brazilian Association of Market Research Companies (ABEP)^l^ (Social Classes A or B; C; D or E), categorized household income in minimum wages (≤ 1; 2-3; 4-5; 6-9; ≥ 10), educational attainment in years of schooling (none; up to three years; four to seven years, eight to 10 years; 11 or more) and occupational status (working; retired or receiving the social security disability insurance; retired and working; homemaker or unemployed). Demographic variables were sex (male; female), age in years according to four categories (60-64; 65-69; 70;-79; ≥ 80), marital status (with a partner; without a partner) and self-reported skin color/race (white; nonwhite).

To describe the sample according to morbidities, the medical diagnosis reported by the respondent was characterized as: diabetes, hypertension, respiratory diseases (asthma; bronchitis; emphysema), rhinitis, glaucoma, Parkinson’s disease, dyslipidemia, and osteoporosis. The need for using long-term medication was assessed by the question “Do you need to take any long-term medication? Consider long-term medication as those you use regularly without a date to stop”. The number of medications used by the older adult in the last 15 days (1, 2, 3, and ≥ 4) was also assessed. Social support was considered present if older adults had someone to help with their needs.

Thirteen trained interviewers visited the selected households to deliver a study presentation letter explaining it and inviting older adults to participate. After eligible individuals’ acceptance, interviews were scheduled according to their availability. Electronic questionnaires with precoded questions were applied using netbooks.

We considered as losses and refusals the interviews not conducted after three attempts, one of those by a study supervisor. Quality control was performed by the field supervisor with 10.0% of respondents selected at random. We used a shorter version of the questionnaire, composed of 19 questions. Correlation was analyzed by the kappa index.

Analyses were performed in the Stata 12.1 program. The sample was described in relation to the independent variables, and the prevalence of the outcome “use of the PFPB-E” was estimated with the respective confidence intervals for individuals who had used any medication in the last 15 days. To estimate the prevalence of reasons for not using the program, we utilized a stratified analysis by age.

The factors associated with the use of PFPB-E were analyzed by Poisson regression, using the *svy* command to consider the study design effect. Variables with p > 0.2 not were taken to adjusted analysis. Regression followed a hierarchical model of backward elimination, which comprises three levels. The distal level included demographic and socioeconomic variables; the second level, the presence of morbidities; and the proximal level, knowledge of the PFPB and the number of medications used. Wald tests were used for heterogeneity and linear trend for the categorical variables. Variables with p < 0.2 were maintained in the model to control confounding factors. The significance level of 0.05 was considered.

To analyze the prevalence of PFPB-E use and associated factors, the total number of older adults who had used medication in the last 15 days was used as a denominator. To characterize medications (presence in the PFPB-E, classification by pharmacological group, gratuitousness of medications), total medications used were the denominator.

This study was approved by the Research Ethics Committee of the Faculdade de Medicina of Universidade Federal de Pelotas (Opinion 472,357). All participants signed an informed consent form and were assured of the confidentiality of information given.

## RESULTS

We interviewed 1,451 older adults. Losses and refusals totaled 21.3% (n = 393), most of them women and aged 60-69 years. Of the interviews, 4.0% were answered by a caregiver or companion. Most older adults (90.5%) had used at least one medication in the last 15 days. Among them, mean age was 70.9 years (SD = 8.2), 64.8% were women, 84.2% were white, 52.4% lived with a partner, and 30.2% had four to seven years of schooling. The predominant occupational status was retired or receiving the social security disability insurance (72.0%), 42.7% had a household income of two to three minimum wages, and 51.8% were part of social class C (Table 1).

**Table 1:** Description of the sample of older adults who had used medications in the last 15 days according to demographic and socioeconomic variables stratified by age. Pelotas, RS, Southern Brazil, 2014. (N = 1,305)

Variable	n^a^	%	95%CI	60-79 years		95%CI	≥ 80 years		95%CI
	
				n	%		n	%	
Age									
60-64	345	26.5	24.1–28.9	-	-	-	-	-	-
65-69	321	24.7	22.3–27.0	-	-	-	-	-	-
70 -79	418	32.1	29.6–34.7	-	-	-	-	-	-
≥ 80	217	16.7	14.6–18.7	-	-	-	-	-	-
Sex									
Male	459	35.2	32.6–37.8	388	35.8	32.9–38.6	70	32.3	25.9–38.5
Female	846	64.8	62.2–67.4	696	64.2	61.3–67.0	147	67.7	61.4–74.0
Marital status									
Living without a partner	620	47.6	44.9–50.3	468	42.6	39.6–45.5	158	72.8	66.8–78.7
Living with a partner	682	52.4	49.7–55.1	624	57.4	54.4–60.3	59	27.2	21.2–33.1
Skin color/Race									
Nonwhite	205	15.8	13.8–17.7	167	15.2	13.1–17.3	40	18.4	13.2–23.6
White	1,097	84.2	82.3–86.2	925	84.8	82.6–86.9	177	81.6	76.3–86.7
Educational attainment (in years of schooling)									
0	176	13.6	11.7–15.5	120	11	9.16–12.9	57	26.6	20.7–32.6
≤ 3	288	23.1	20.7–25.3	244	22.5	20.0–24.9	56	26.2	20.2–32.1
4-7	391	30.2	27.7–32.7	339	31.2	28.3–33.8	56	26.2	20.2–32.1
8-10	132	10.2	8.5–11.8	122	11.2	9.4–13.2	10	4.7	1.8–7.5
≥ 11	296	22.9	20.6–25.2	261	24.1	21.6–26.7	35	16.3	11.3–21.3
Occupational status									
Working	89	7.4	5.9–8.8	90	8.8	6.9–10.4	1	0.5	–0.05–1.5
Retired or receiving the social security disability insurance	871	72.0	69.5–74.6	705	69.2	66.3–72.0	169	87.1	82.3–91.8
Retired and working	125	10.3	8.6–12.1	117	11.2	9.4–13.3	10	5.2	2.0–8.2
Homemaker or unemployed	124	10.3	8.5–11.9	111	10.8	8.9–12.7	14	7.2	3.5–10.9
Economic classification - ABEP									
A or B	449	36.4	33.7–39.1	373	36.3	33.3–39.2	76	36.7	30.0–43.3
C	639	51.8	49.0–54.6	544	52.9	49.8–55.9	98	47.3	40.5–54.2
D or E	145	11.8	9.9–13.5	111	10.8	8.9–12.7	33	16.0	10.9–21.0
Income in minimum wages									
≤ 1	125	10.3	8.6–12.0	105	10.4	8.4–12.2	19	9.4	5.3–13.5
2-3	519	42.7	39.8–45.4	445	43.7	40.6–46.7	77	38.3	31.5–45.1
4-5	257	21.1	18.8–23.4	207	20.3	17.8–22.8	51	25.4	19.3–31.4
6-9	177	14.5	12.6–16.5	142	13.9	11.9–16.2	35	17.4	12.1–22.7
≥ 10	139	11.4	9.6–13.2	119	11.7	9.6–13.6	19	9.5	5.3–13.5
Social support^b^									
No	96	7.4	6.0–8.8	84	7.8	6.2–9.4	11	5.1	6.1–9.4
Yes	1,205	92.6	91.2–94.0	997	92.2	90.6–93.8	205	94.9	90.6–93.8
Knowledge of the PFPB									
No	165	12.6	10.8–14.5	101	9.3	7.6–11.0	62	28.7	22.6–34.8
Yes	1,139	87.4	85.5–89.1	984	90.7	88.9–92.4	154	71.3	65.2–77.4
Use of the PFPB-E									
No	534	43.2	40.4–45.9	408	39.8	36.8–42.8	123	58.8	52.1–65.6
Yes	703	56.8	54.1–59.6	616	60.2	57.2–63.2	86	41.2	34.4–47.9
Number of medications used									
1	163	12.5	10.7–14.3	144	13.3	11.2–15.3	17	7.8	4.2–11.4
2	203	15.6	13.6–17.5	174	16.1	13.8–18.2	28	12.9	8.4–17.4
3	234	17.9	15.8–20.0	200	18.4	16.1–20.8	33	15.2	10.4–20.0
≥ 4	705	54.0	51.3–56.7	566	52.2	49.2–55.2	139	64.1	57.6–70.5
Need for long-term medication									
No	68	5.2	4.0–6.4	60	5.5	4.2–6.9	8	3.7	1.1–6.2
Yes	1,237	94.8	93.6–96.0	1,024	94.5	93.1–95.8	209	96.3	93.8–98.8
Health conditions variables									
Diabetes									
No	973	74.6	72.3–76.9	802	73.9	71.4–76.6	169	77.9	72.3–83.4
Yes	331	25.4	23.0–27.7	282	26.1	23.4–28.6	48	22.1	16.6–27.7
Hypertension									
No	371	28.5	26.0–30.9	309	28.7	26.0–31.5	61	28.2	22.2–34.3
Yes	933	71.5	69.1–74.0	766	71.3	68.5–73.9	155	71.7	65.7–77.8
Chronic respiratory diseases									
No	1,077	82.7	80.7–84.8	905	83.6	81.4–85.8	169	77.9	72.3–83.4
Yes	225	17.3	15.2–19.3	177	16.4	14.2–18.6	48	22.1	16.5–27.6
Rhinitis									
No	1,037	79.7	77.5–81.9	857	80.2	77.8–82.6	177	82.7	77.6–87.8
Yes	264	20.3	18.1–22.5	211	19.8	17.4–22.1	37	17.3	12.2–22.4
Glaucoma									
No	1,201	92.3	90.9–93.8	1,010	93.3	91.9–94.8	188	88.3	83.9–92.6
Yes	100	7.7	6.2–9.1	72	6.7	5.2–8.1	25	11.7	7.3–16.1
Parkinson’s disease									
No	1,280	98.3	97.6–99.0	1,068	98.7	98.0–99.4	209	96.3	93.8–98.9
Yes	22	1.7	0.9–2.3	14	1.3	0.06–1.9	8	3.7	1.1–6.2
Dyslipidemia									
No	733	56.2	53.5–58.9	594	55.3	52.3–58.3	137	63.7	52.2–70.2
Yes	570	43.8	41.0–46.4	480	44.7	29.8–42.7	78	36.3	29.8–42.7
Osteoporosis									
No	954	73.3	70.9–75.7	809	75.5	72.9–78.0	142	65.7	59.4–72.1
Yes	347	26.7	24.3–29.1	263	24.5	21.9–27.1	74	34.3	27.9–40.6

ABEP: Brazilian Association of Market Research Companies; PFPB: Brazilian People’s Pharmacy Program; PFPB-E: Brazilian People’s Pharmacy Program – expansion model

^a^ The maximum number of ignored values was 96, for occupational status.

^b^ Social support: possibility of the older adult.

About of 93.0% of the older adults reported having someone to help them with their needs. Regarding self-reported health conditions, 25.4% had diabetes, 71.5% hypertension, and 17.3% respiratory diseases (Table 1).

The prevalence of PFPB-E use was 56.8% whilst the prevalence of knowledge of the program was 87.4%. About of 95.0% of the older adults had to take long-term medication, and 54.0% used four or more medications (Table 1). Of the individuals reporting long-term use of medication and taking medication in the last 15 days, 53.9% used the PFPB-E to obtain at least one medication (data not shown in tables). Considering age stratification, 71.3% of the people aged 80 years or over knew the PFPB, 41.2% had used the program and 64.1% used four or more medications (Table 1).

Among the 1,014 older adults who had not acquired all their medications from the PFPB-E or directly from SUS units, 54.0% had tried obtaining them from the program. The main reasons reported for not having obtained medication from the program were the medication not being in the PFPB-E list (74.0%), difficulty in getting the prescription (17.0%), and medication shortage (13.0%). Among those who did not try to get the medication from the PFPB-E, the main reasons were the medication not being in the PFPB-E list (46.0%), ignorance of which medications were available (39.0%) and no knowledge of the PFPB (29.0%) (Table 2).

**Table 2:** Reasons for not using the Brazilian People’s Pharmacy Program, by older adults who had not obtained all their medications from the PFPB-E or SUS in the last 15 days, according to age. Pelotas, RS, Southern Brazil, 2014. (N = 1,014)

Variable	General		Age group			

			60-79		≥ 80	
		
	n	%	n	%	n	%
Tried to obtain a medication from the PFPB-E						
No	462	45.6	356	42.8	105	58.3
Yes	552	54.1	476	57.2	75	41.7
Reasons for trying and failing (n = 552)						
Difficulty in going to the pharmacy	31	5.7	20	4.2	9	12.3
Shortage of medication of the prescribed brand	49	8.9	41	8.7	8	10.8
Medication shortage	69	12.6	59	12.5	10	13.5
Difficulty in obtaining the prescription	93	17.0	77	16.3	16	21.6
Medication is not in the PFPB list	408	74.4	349	73.9	57	77.0
Other	43	7.8	38	8.0	5	6.8
Reasons for not trying (n = 469)						
Not trusting PFPB medication	15	3.4	13	3.8	2	2.0
A physician advised against it	16	3.6	13	3.8	3	3.0
Difficulty in going to the pharmacy	34	7.6	18	5.2	16	16.2
Difficulty in obtaining the prescription	55	12.3	43	12.5	12	11.9
Not knowing the PFPB	128	28.6	85	24.6	43	42.2
Not knowing which medications are available in the PFPB	174	39.1	127	36.9	47	47.0
Medication is not in the PFPB list	185	45.7	155	48.6	30	35.3
Other reasons	84	18.9	67	19.5	17	16.8

PFPB-E: Brazilian People’s Pharmacy Program – expansion model; SUS: Brazilian Unified Health System; PFPB: Brazilian People’s Pharmacy Program

Of the people aged 80 years or over who had not obtained all their medications from the PFPB-E or SUS, 42.0% had tried to get the medications from the program. This age group had more difficulty in obtaining the prescription (21.0%) and going to the pharmacy (12.0%) than the 60-79 years group (16.0% and 4.0%, respectively). About 45.0% of them reported lacking knowledge of the program and medications available as a reason not to try obtaining the medication from the PFPB-E. Among older people aged 80 years or over, 16.0% reported difficulty in going to the pharmacy as a reason (Table 2).

Table 3 describes the factors associated with the use of the PFPB-E. The crude analysis showed no association with sex, skin color/race, occupation and social support. People aged 80 years or older presented a 28.0% lesser use of the PFPB-E than those aged 60-64 years, while those living with partners used it 13.0% more than those without a partner. The use of the program decreased linearly with increasing income, and individuals with household income equal to or greater than 10 minimum wages used the program 34.0% less than those of lower income.

**Table 3:** Factors associated with use of the People’s Pharmacy Program in the last 15 days. Pelotas, RS, Southern Brazil, 2014.

Level^a^	Variable	p	95%CI	PR_crude_	95%CI	p	PR_adjusted_	95%CI	p^b^
1	Age					< 0.001			0.004
	60-64	60.6	34.0–44.7	1	-	-	1	-	-
	65-69	59.1	53.5–64.7	0.97	86.3–1.10	-	0.97	0.85–1.11	-
	70-79	60.5	55.7–65.3	0.99	87.7–1.14	-	1.02	0.89–1.16	-
	≥ 80	41.1	34.4–47.9	0.67	56.3–81.9	-	0.72	0.60–0.87	-
	Educational attainment (in years of schooling)					0.007			0.131
	0	48.0	40.4–55.5	1	-	-	1	-	-
	≤ 3	60.8	55.0–66.5	1.26	1.03–1.55	-	1.22	0.99–1.52	-
	4-7	63.1	58.2–68.0	1.31	1.06–1.62	-	1.31	1.06–1.62	-
	8-10	56.8	47.7–65.8	1.18	0.93–1.50	-	1.20	0.94–1.54	-
	≥ 11	50.2	44.3–56.1	1.05	0.84–1.30	-	1.14	0.90–1.46	-
	Income in minimum wages					0.001^c^			0.005^c^
	≤ 1	56.9	47.7–66.0	1	-	-	1	-	-
	2-3	61.5	57.2–65.8	1.08	0.90–1.30	-	1.0	0.83–1.20	-
	4-5	60.0	53.8–66.2	1.05	0.86–1.30	-	0.97	0.77–1.22	-
	6-9	50.6	43.0–58.1	0.88	68.7–1.15	-	0.83	0.63–1.10	-
	≥ 10	40.9	32.4–49.4	0.72	0.55–0.94	-	0.66	0.49–0.90	-
	Marital status					0.013			0.036
	Living without a partner	52.6	48.5–56.6	1	-	-	1	-	
	Living with a partner	60.8	57.0–64.6	1.16	1.03–1.30	-	1.13	1.0–1.28	
2	Hypertension					< 0.001			< 0.001
	No	38.0	32.6–43.4	1	-	-	1	-	
	Yes	63.2	60.0–66.3	1.66	1.41–1.96	-	1.52	1.29–1.77	
	Diabetes					< 0.001			< 0.001
	No	52.7	49.4–55.9	1	-	-	1	-	
	Yes	68.1	63.0–73.1	1.30	1.19–1.40	-	1.19	1.09–1.30	
	Dyslipidemia					< 0.001			< 0.001
	No	48.1	44.3–51.8	1	-	-	1	-	
	Yes	67.3	63.4–71.1	1.40	1.27–1.54		1.31	1.20–1.43	
	Glaucoma					0.128			0.129
	No	56.2	53.4–59.1	1			1		
	Yes	63.9	54.2–73.6	1.14	0.96–1.34		1.13	0.96–1.34	
	Rhinitis					0.050			0.05
	No	55.4	52.3–58.5	1	-	-	1		
	Yes	62.1	56.0–68.2	1.12	1.0–1.25	-	1.12	0.99–1.26	
3	Knowledge of the PFPB					< 0.001			< 0.001
	No	16.4	10.2–22.6	1	-	-	1	-	-
	Yes	61.9	59.0–64.8	3.77	2.63–5.40	-	2.87	2.01–4.08	-
	Number of medications used					< 0.001^c^			< 0.001^c^
	1	37.5	28.7–46.3	1	-	-	1	-	-
	2	41.1	34.0–48.1	1.09	80.3–1.49	-	0.96	71.6–1.28	-
	3	53.5	46.9–60.0	1.42	1.06–1.90	-	1.22	92.0–1.62	-
	≥ 4	65.6	62.0–69.1	1.74	1.35–2.26	-	1.40	1.07–1.80	-

PFPB: Brazilian People’s Pharmacy Program

^a^ All variables were controlled for the others of the same level and above, with p from 0.2 to 0.05.

^b^ The variables with significance level from 0.05 to 0.2 were maintained in the model to control confounding.

^c^ Wald Test for linear trend.

Individuals with chronic diseases as diabetes, hypertension, and dyslipidemia used the program 19.0%, 52.0%, and 31.0% more, respectively, than those without these morbidities. Older people who knew the PFPB used it three times more than those who did not, and individuals who took four or more medications used the PFPB-E 40.0% more than those who took only one medication (Table 3).

Of the 5,700 medications used by the older adults, 32.9% (n = 1,873) were part of the PFPB-E; of these, 67.6% (n = 1,249) were obtained from the program, of which 79.3% (n = 991) free of charge. As to the origin of the prescription used to obtain the medications from the people’s pharmacy, 54.4% were from private or health plan professionals. The most used medications by the PFPB-E, according to pharmacological classification, were antihypertensive drugs (58.3%), antidiabetic drugs (18.7%), and lipid modifying agents (17.5%) (Table 4).

**Table 4:** Description of the medications in the PFPB-E list according to use of the program. Pelotas, RS, Southern Brazil, 2014. (N = 1,873)

Medication obtained from the PFPB-E	n	%
No	599	32.4
Yes	1,249	67.6
Medications obtained free of charge from the PFPB-E		
No	258	20.7
Yes	991	79.3
Origin of the prescription of the medications obtained from the PFPB-E		
SUS	569	45.6
Health plan or private	678	54.4
Description of the PFPB-E medications according to pharmacologic class		
Respiratory diseases (asthma, bronchitis, emphysema)	31	1.7
Diabetes	351	18.7
Dyslipidemia	327	17.5
Glaucoma	15	0.8
Hypertension	1,092	58.3
Osteoporosis	34	1.8
Parkinson’s disease	13	0.7
Rhinitis	10	0.5

PFPB-E: Brazilian People’s Pharmacy Program – expansion model; SUS: Brazilian Unified Health System

Among the medications used for chronic conditions such as respiratory diseases, diabetes, and hypertension, 41.9%, 85.2%, and 58.9%, respectively, were present in the PFPB-E list (data not shown in tables).

The design effect of this study was 1.14, the intra-class correlation coefficient was 0.0099, and the kappa index of the variable “PFPB knowledge” presented good reproducibility (kappa ≥ 0.7).

## DISCUSSION

Of the older people who used medications, more than 80.0% knew the PFPB and more than half used it. However, those aged 80 years or over used the program less. This shows that the program is very disseminated, except among the oldest adults. In addition, a significant portion of the older adults with prescriptions from SUS obtained medications from the PFPB-E, even though they are not its priority group.

In a 2008 population-based study in adults aged 20 years or over, knowledge of the PFPB was 32.8%^8^. The greater knowledge of PFPB in this study may result from the increased time between data collection and the beginning of the program or from the exclusive participation of older adults, who use medications and health services more, increasing the opportunities to learn about the program^8^ ^,^ ^10^.

The main barriers to the use of the program are lack of prescription, medication shortage, and the medication not being part of the program, suggesting difficulty of access to medical appointments and insufficient programming of medication supply and distribution. Although the number of medications offered by the program is great (including the vast majority of treatments for chronic conditions) and the list is revised periodically, it is necessary to assess whether the medications included in the program satisfy properly the treatment needs of the population^9^. On the other hand, the program includes medications similar to others that are not part of the program, indicating that physicians possibly fail to prioritize or to know the medications in the program^1^ ^,^ ^16^.

We also noted that difficulty in going to the pharmacy is a major problem for people aged 80 years or over since, in the absence of the patient, a legal representative is required to collect the medication. Although the existence of the program is widely known, there is still a lack of information, especially among people aged 80 years or over. The list of medications offered by the program is the least known aspect by the population.

Use of the PFPB-E was greater in the older adults aged less than 80 years. The reduction in the use of the program by people aged 80 years or over might reflect inequality in access to medications since as age increases, older adult tends to be more medicalized. This inequality might be related to inability or difficulty of mobility, which hinders access to medical appointments to renew the prescription and going to the registry to obtain the power of attorney required in case of a legal representative collecting the medication.

The study was consistent with the findings of Costa et al.^8^, who found no association between sex of respondents and use of the program. Women live longer, seek health services more^2^ and use more medications^2^ ^,^ ^10^ ^,^ ^18^. Thus, no association can be observed for the concentration of women in the 80 years or over group, which faces barriers to access the program.

Individuals with partners used the PFPB-E more, which is consistent with the fact that individuals with partners use health services more^7^ ^,^ ^8^. Consequently, these individuals are more likely to diagnose diseases, to have access to prescriptions and to receive information regarding the PFPB.

This study shows, consistently with the literature, that the program serves all socioeconomic strata of the population^8^ ^,^ ^15^. In addition, although most prescriptions used by the elderly to obtain the medications were from the private sector, the number of users of the public network is high, accounting for 46.0%, in accordance with what Pinto et al.^14^ ^,^ ^15^ found in their research.

A 2007 case study including municipalities from the five regions of Brazil suggests that users may have sought the program because of the ready availability of medications and its shorter wait. Centralizing medication purchasing can reduce pharmaceutical assistance costs, but the fact that medications for asthma, diabetes, and hypertension are available free of charge in the PFPB does not exempt state and municipal managers from the continuity of their provision. They should be available in primary health care pharmacies, facilitating the access for SUS users^m^. Thus, failures in the free-of-charge provision of medications by municipal public networks overloads the demand of the PFPB-E, creating costs for the user, both with medications not covered by the program and with transportation to the pharmacy.

Medication shortage in the public and private sectors (PFPB-E) implies a greater part of users’ income destined to health expenditure, which is already extremely high. The situation is even more complicated for those who fail to adhere to the treatment for the lack of money to purchase medication, which often worsens their health status^4^.

In accordance with previous studies^6^ ^,^ ^11^ ^,^ ^15^ ^,^ ^17^, the most used PFPB medications were those for cardiovascular conditions and those related to metabolism. That can be partly explained by the fact that these medications are available free of charge and by their consumption pattern, which reflects the profile of the chronic diseases most prevalent among older adults^5^ ^,^ ^12^.

The studied sample is representative of the older population residing in urban areas. Losses were not substantially different from the studied population, which reinforces the internal validity. As this study addressed only the PFPB private networks, its results can be generalized to municipalities with these characteristics. To better qualify the outcome characterization, we requested the packaging or prescription of all medications listed by the respondents. The lack of other household surveys concerning the PFPB-E limited the consistency analysis of this study.

The results obtained by this population survey expand knowledge regarding the use of the PFPB-E by the older population and of the reasons for not using it. Thus, they contribute to defining policies to qualify the program. People aged 80 years or over present a great need for access to medications and, according to the Brazilian Institute of Geography and Statistics (IBGE), the number of people in this age group increased by 70.0% from 2000 to 2010^n^. This situation requires measures to reduce the inequality in the pharmaceutical assistance to this age group as well as to expand the use of the program. Expanding home care can contribute to this end, facilitating the obtaining of the prescription. However, considering the mobility difficulties characteristic of this population group, it is critical to study alternatives to the requirement of power of attorney for legal representatives to receive medication from the pharmacy.

Disseminating the list of medications offered by the program both for the population and for physicians is necessary. This knowledge can contribute to increasing the use of the program by older adults and to health professionals, whenever possible, directing the prescription to medications in the list, reducing expenditure on long-term medications, which are especially important for this population^1^ ^,^ ^9^.

The PFPB added an enormous cost to pharmaceutical assistance with the currently available medications and the gratuitousness of the most used medications for asthma, diabetes and hypertension. A PFPB medication can cost up to 290.0% more for the Brazilian Ministry of Health than for the *Farmácia Básica* (Primary Pharmacy)^o^.

It is crucial that local authorities take responsibility for the pharmaceutical assistance of SUS users, promoting access to medication and the effective insertion of pharmaceutical assistance as a health action, especially for those in remote areas, with a medication supply closer to the user. This strategy is crucial to avoid spending on medications and transportation, and even low adherence to treatment in the low-income population.

## Footnotes

a Ministério da Saúde, Secretaria de Políticas de Saúde. Política Nacional de Medicamentos: informe técnico institucional. Brasília (DF); 2000.

b Ministério da Saúde. Programa Farmácia Popular do Brasil: manual básico. Brasília (DF); 2005.

c Ministério da Saúde, Secretaria de Ciência, Tecnologia e Insumos Estratégicos, Departamento de Assistência Farmacêutica e Insumos Estratégicos. Programa Farmácia Popular do Brasil: manual de informações às unidades credenciadas: sistema de co-pagamento. 2.ed. Brasília (DF); 2008.

d Brasil. Decreto nº 5.090, de 20 de maio de 2004. Regulamenta a Lei nº 10.858, de 13 de abril de 2004, e institui o programa “Farmácia Popular do Brasil” e dá outras providências. Brasília (DF); 2004 [cited 2015 Sept 16]. Available from: http://www.planalto.gov.br/ccivil_03/_ato2004-2006/2004/decreto/d5090.htm

e Ministério da Saúde. Portaria nº 749, de 15 de abril de 2009. Dispõe sobre a expansão do Programa Farmácia Popular do Brasil - Aqui Tem Farmácia Popular. Brasília (DF); 2009 [cited 2015 Sept 16]. Available from: http://bvsms.saude.gov.br/bvs/saudelegis/gm/2009/prt0749_15_04_2009.html

f Portal Brasil. Gratuidade de medicamentos beneficia 14 milhões de pessoas. Brasília (DF); 2013 [cited 2013 Mar]. Available from: http://www.brasil.gov.br/governo/2013/02/gratuidade-de-medicamentos-beneficia-14-milhoes-de-pessoas.

g Ministério da Saúde. Portaria nº 1.146, de 1º de junho de 2012. Altera e acresce dispositivos à Portaria nº. 971/GM/MS, de 17 de maio de 2012, para ampliar a cobertura da gratuidade no âmbito do Programa Farmácia Popular do Brasil. Brasília (DF); 2012 [cited 2015 Sept 16]. Available from: http://bvsms.saude.gov.br/bvs/saudelegis/gm/2012/prt1146_01_06_2012.html

h World Health Organization. Envelhecimento ativo: uma política de saúde. Brasília (DF): Organização Pan-Americana da Saúde; 2005 [cited 2015 Sept 16]. Available from: http://bvsms.saude.gov.br/bvs/publicacoes/envelhecimento_ativo.pdf

i Ferreira RL. Análise de implantação do Programa Farmácia Popular do Brasil no Rio de Janeiro e no Distrito Federal: um estudo de casos [dissertation]. Rio de Janeiro (RJ): Escola Nacional de Saúde Pública Sergio Arouca da Fundação Oswaldo Cruz; 2006.

j Junges F. Avaliação do Programa Farmácia Popular do Brasil: aspectos referentes a estrutura e a processos [dissertation]. Porto Alegre (RS): Faculdade de Farmácia da Universidade Federal do Rio Grande do Sul; 2009.

k Instituto Brasileiro de Geografia e Estatística. Censo demográfico 2010: Cidades: Rio Grande do Sul: Pelotas. Rio de Janeiro; s.d.[cited 2014 Dec 5]. Available from: http://cidades.ibge.gov.br/painel/painel.php?lang=&codmun=431440&search=|pelotas

l Associação Brasileira de Empresas de Pesquisa - ABEP. Critério de Classificação Econômica Brasil - CCEB 2012: Base LSE 2010. Available from: http://www.abep.org/criterio-brasil

m Ministério da Saúde. Crescimento do Programa Farmácia Popular. Brasília (DF); 2013 [cited 2014 Mar]. Available from: http://www.cosemsms.org.br/ultimas-noticias/farmacia-popular-nota-de-esclarecimento

n Instituto Brasileiro de Geografia e Estatística. Censo demográfico e contagem da população. Rio de Janeiro (RJ); 2014. Available from: http://www.sidra.ibge.gov.br/bda/tabela

o Carraro WBWH. Desenvolvimento econômico do Brasil e o Programa Aqui Tem Farmácia Popular: limitantes e potencialidades [these]. Porto Alegre (RS): Faculdade de Ciências Econômicas da UFRGS; 2014.
